# Identification of Hot and Cold Regions for Mutagenesis in the *Escherichia coli tdk* Gene: A Catalog of Mutational Sites

**DOI:** 10.1002/em.70021

**Published:** 2025-06-22

**Authors:** Katherine Douglas, Dana Sorensen, Arnav Saud, Ananya Sridharan, Mallika Mathew, Jyotsna Hiranandani, Ava Gonick, Courtney Young, Kelly Nguyen, Nhu Phi, Jeffrey H. Miller

**Affiliations:** ^1^ Department of Microbiology, Immunology, and Molecular Genetics, and the Molecular Biology Institute University of California, and the David Geffen School of Medicine Los Angeles California USA

**Keywords:** bacterial systems, mutational hotspots, *tdk*

## Abstract

We constructed a catalog of mutational sites in the *tdk* gene of *Escherichia coli* that consists of 378 different base pair substitutions at 245 different sites (base pairs). This allows us to examine the tendency of different sub‐regions of the gene to be more or less prone to mutations when compared with other sub‐regions. We do this by recording the number of occurrences of close to 1100 mutations resulting from 9 different mutagens or mutators at each site within a rolling 20‐site interval. We can define 5 mutationally prone regions (MPRs). Aligning the profile of MPRs in *tdk* next to those we previously described in the t*hyA* gene results in a linear map with 8 MPR regions, reinforcing the concept that certain sub‐regions of genes are more mutable than others, even when other variables are accounted for. The catalog should prove useful for future studies.

## 1 Introduction

Hotspots, sites in a gene that are dramatically more mutable than other sites, have been the object of intense study since they were first described by Benzer (1961). Although the preference for repeated sequence elements is clearly crucial for creating frameshifts and in‐frame short deletions and additions (Streisinger et al. 1966; Farabaugh et al. 1978; Pribnow et al. 1981), all the rules for determining base substitution hotspots have yet to be elucidated. In some cases, special mechanisms exist, such as hotspots at 5‐methylcytosines (Coulondre and Miller 1977) or template switching at an inverted repeat at a secondary structure in the *thyA* gene (Viswanathan et al. 2000). More generally, nearest neighbor bases clearly play a role in determining base substitution mutation rates (Coulondre and Miller 1977; Miller 1985; Pienkowska et al. 1993) as also revealed by multiple additional studies. For example, whole genome sequencing in the case of mismatch repair deficient *E. coli* (Foster et al. 2018). Such context effects have been shown for furazolidine hotspots in *lacI* (Bertenyi and Lambert 1996), for alkylating agents in *lacI* (Horsfall et al. 1990), for benzo[a]pyrene diol epoxide in the *supF* gene (Rodriguez and Loechler 1991), for uracil removal from DNA (Nilsen et al. 1995), for mutations resulting from strand breaks (Shee et al. 2012), and from oxidative damage (Hatahet et al. 1998). Nearest neighbor preferences are the basis for the “mutational signatures” seen in genomic sequencing analysis of mammalian genomes (e.g., Phillips 2018). Yet even among sites with identical nearest neighbors, mutation rates can vary as much as two orders of magnitude (e.g., Miller 1985; Pienkowska et al. 1993; see Discussion in Fernandez et al. 2020). Mutational hotspots are not confined to bacterial and phage systems. For example, recent work with cancer genomes has analyzed mutational hotspots in coding and non‐coding regions (Piraino and Furney 2017; see also Lawrence et al. 2013).

We previously analyzed mutational sites in the *E. coli thyA* gene, and showed that some regions of the gene were more prone to mutations at known base substitution sites than other regions (Mashiach et al. 2021). Analyzing over 1100 mutations from different treatments and mutator strains, we defined three “MPRs” (mutationally prone regions). To prove more definitively that MPRs play a vital role in determining hotspots, we sought to repeat this analysis using a single mutagenic treatment, and also to look for MPRs in a different gene, as we need to expand the number of MPRs beyond the three that were found in *thyA*. We subsequently developed a second gene‐reporter system, the *tdk* gene that encodes thymidine deoxykinase, as mutants resistant to AZT (3′‐azido‐3′‐deoxythymidine) result from defects in the *tdk* gene (e.g., Young et al. 2024). Developing new reporter systems is important, as adding more sequence contexts to the overall examination of mutational preferences offers a more complete picture than any one system. For example, the *tdk* gene is AT rich compared to other gene reporter systems in *E. coli* . Moreover, mutants are collected on rich media containing AZT plates after just 18–24 h. The relatively small size (618 bp) of the t*dk* gene allows for sequencing the whole gene with a single sequencing primer. Using the *tdk* system, we showed that some of the hotspots for a specific mutagen, cisplatin (CPT), occurred only in one region of the gene, even though verified sites with identical nearest neighbors were available in other parts of the gene (Young et al. 2024). Here, we describe an extensive cataloging of the mutational sites in that *tdk* gene that result in a defective enzyme via specific base substitutions and use this catalog to analyze more than 1100 mutations detected from 9 different mutagenic treatments or “mutator strains” with specific repair defects. These are: 2‐aminopurine (2AP), ethyl methanesulfonate (EMS), 4‐nitroquinoline‐1‐oxide (NQO), cisplatin (CPT), 5‐azacytidine (5AZ), *mutD, mutS, mutT*, and *mutY*. The resulting catalog defines 378 different mutations at 245 distinct sites (base pairs) in the *tdk* gene. This allows us to define 5 MPR's in the gene and extends the analysis of Mashiach et al. (2021) to a second gene‐protein system. Together, these two studies show 8 sub‐regions of genes that are mutationally “hot” and corresponding sub‐regions that are mutationally cold. These regions are always normalized to the same number of available known sites and are thus not an artifact resulting from critical regions of the corresponding protein.

## 2 Materials and Methods

### 2.1 *E. coli* Strains

The wild‐type strain is BW25113 (Datsenko and Wanner 2000) that we have used previously (e.g., Ang et al. 2016; see below). It is the starting strain for the KEIO collection, described in (Baba et al. 2006). This strain is *lacI* ^*q*^ *rrnB*_*T14*_ *ΔlacZ*_*WJ16*_ *hsdR514 ΔaraBAD*_*AH33*_ *ΔrhaBAD*_*LD78*_. The MutT, MutY, MutD, and MutS‐deficient strains used here are from the Keio collection.

### 2.2 Media

The following media (Miller 1972, 1992) were used. LB (10 g tryptone, 5 g yeast extract,10 g NaCl per liter). Minimal A buffer 10.5 g K_2_HPO_4_, 4.5 g KH_2_PO_4_, 1 g (NH_4_)_2_SO_4_, 0.5 g sodium citrate·2H_2_O per liter.

### 2.3 Growth Conditions, Mutagenesis Treatment

Unless otherwise stated, all genetic methods are as described by Miller (1972, 1992).

NQO (4‐nitroquinoline‐1‐oxide), EMS (ethyl methanesulfonate), CPT (cisplatin) treatment: Overnight cultures grown in a 37° incubator were used to seed “overday” cultures by inoculating 250 μL into each of 5 separate 5 mL LB cultures that were then incubated in a 37° water bath for 3–4 h. After placing on ice for 5 min, the cultures were pooled and spun down, the liquid discarded, and the pellets washed in minimal A buffer (nonsupplemented minimal A), recentrifuged, and resuspended in half the initial LB volume in minimal A buffer. For NQO, 2 mL samples were aliquoted into clean culture tubes and NQO was added to a final concentration of 50 μg/mL. The NQO was diluted from a freshly prepared stock solution of 5 mg/mL in acetone. Treatment was for 60 min at 37°. For EMS, 0.03 mL EMS was added to 2 mL cultures that were incubated for either 30 min or 60 min. For CPT, CPT was added to a final concentration of 100 μg/mL and incubated for 60 min. (CPT was prepared in a 2 mg/mL stock in solution in water that was shaken vigorously for 1.5 h). In all cases (NQO, EMS, CPT), the cells were spun down and resuspended into 2.5 mL minimal A buffer. These cultures were titered for survival. Outgrowth cultures were seeded with 0.2 mL of this resuspension in 5 mL LB, grown overnight at 37° on a rotor at 50 rpm (0.44 g) before plating. 2AP (2‐aminopurine) and 5AZ (5‐azacytidine) treatment: 2 mL LB cultures containing either 250 or 500 μg/mL 2AP or 22 μg/mL 5AZ were seeded with approximately 1000 cells and grown overnight. The treatments with NQO, CPT, and EMS yielded survivals of 45%–70% (average). Cells growing in 2AP and 5AZ yielded 85%–95% growth of the wild‐type in overnight cultures.

For mutator strains, a culture grown overnight in an incubator at 37°C without additional aeration was used to seed sets of cultures with and without mutagens. We diluted the cultures (10^−4^ dilution) and then added 20 μL (approximately 500–800 cells) to 2 mL cultures with LB. These were then grown overnight at 37°C on a rotor at 50 rpm.

### 2.4 Determination of Mutant Frequencies

The cells grown as indicated above were plated on LB plates with or without 100 ng/mL azidothymidine (AZT; Young et al. 2024). The plates were scored after 24 h. The frequencies of AZT‐resistant mutants were determined as described previously (Young et al. 2024). Briefly, mutant frequency (f) was determined as the median frequency from a set of cultures (*N* = number of cultures). 95% confidence limits were determined according to (Dixon and Massey Jr. 1969). The frequencies are given in Table 1.

**TABLE 1:** Mutant frequencies(f) of *E.coli* using different strains and treatments.

Strain/treatment	Condition (μg/ml)	Number of cultures	Frequency *f* (10^−8^)^a^
BW25113 (WT)	—	16	153 (97–205)
BW25113 4NQO	50	6	3580 (2180‐17,400)
BW25113 5AZ	20	10	8610 (5240‐22,800)
BW25113 2AP	500	14	8060 (5450‐9300)
BW25113 EMS	15	4	49,500 (17,600‐93,000)
Mut D	—	5	566,000 (429,000‐697,000)
Mut T	—	12	12,000 (7160‐22,300)
Mut S	—	9	4490 (1900 – 5810)
Mut Y	—	13	579 (373–1160)

^a^ Values in parentheses are 95% confidence limits.

### 2.5 Chromosomal DNA Isolation and Sequencing

Chromosomal DNA was isolated from overnight cultures of each mutant after single colony purification by streaking. We only picked one AZT‐resistant mutant per culture. The PCR tests were carried out as colony PCR reactions. The *tdk* gene was PCR‐amplified from genomic DNA using Taq polymerase (Invitrogen) and sets of primers which allowed us to sequence directly from the PCR product. The primer sequences were: tdk F (forward) 22mer 5′—CAAGGCTTCGTAAGGGAGAACG—3′, tdk R (reverse) 21mer 5′—CTGCCGAGAAGGGTATATAGC—3′; The sequencing primer was tdk F. The tdk F primer extends from 120 bases to 98 bases upstream of the 5' end of the gene. The reverse primer extends from 72 bases to 93 bases downstream of the 3' end of the gene.

The full PCR procedure is as follows:

In total, there are 3 cycles. The first cycle is done once and holds at the melting temperature of 94.0 C for 2 min. The second cycle is repeated 30 times and has three steps. First, the melting temperature of 94.0 C is held for 30 s, then annealing occurs at 58.0 C for 30 s, followed by extension at 72.0 C for 1 min. The third and final cycle is done once and has two steps. The first step is at the extension temperature of 72.0 C for 7 min, followed by holding at the final dwell temperature of 4.0 C.

The unpurified PCR product was outsourced to Laragen (Culver City) for purification with exoSap (Affymetrix) and Sanger sequencing.

### 2.6 Chemicals

AZT, ethyl methanesulfonate (EMS), nitroquinoline oxide (NQO), 5‐azacytidine (5AZ), cisplatin, and 2‐aminopurine (2AP) were purchased from Sigma (St. Louis, MO).

## 3 Results

### 3.1 Mutational Sites in *Tdk*

**FIGURE 1:**
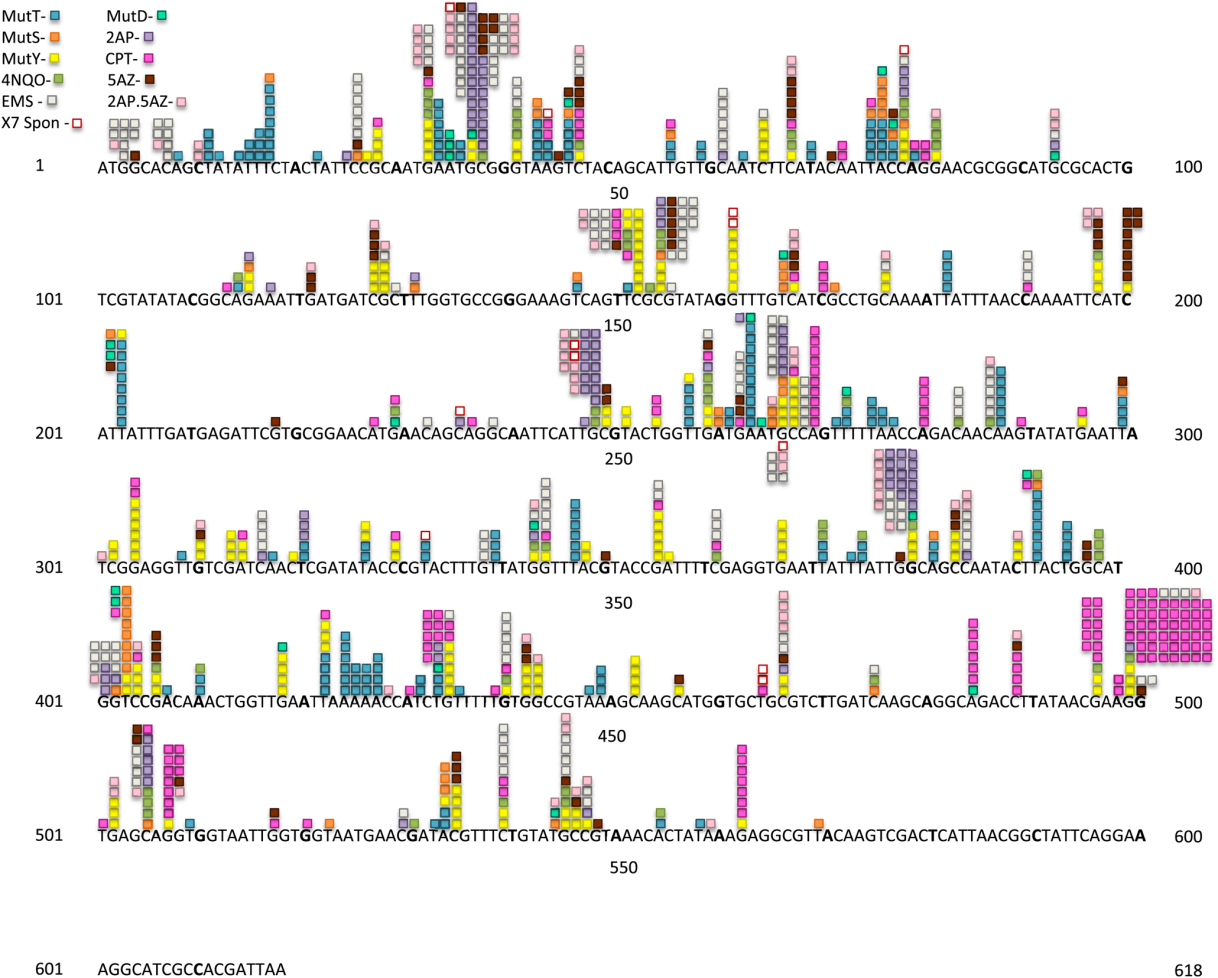
Distribution of 1086 base substitution mutations in the *tdk* gene in strain BW25113.

**FIGURE 2:**
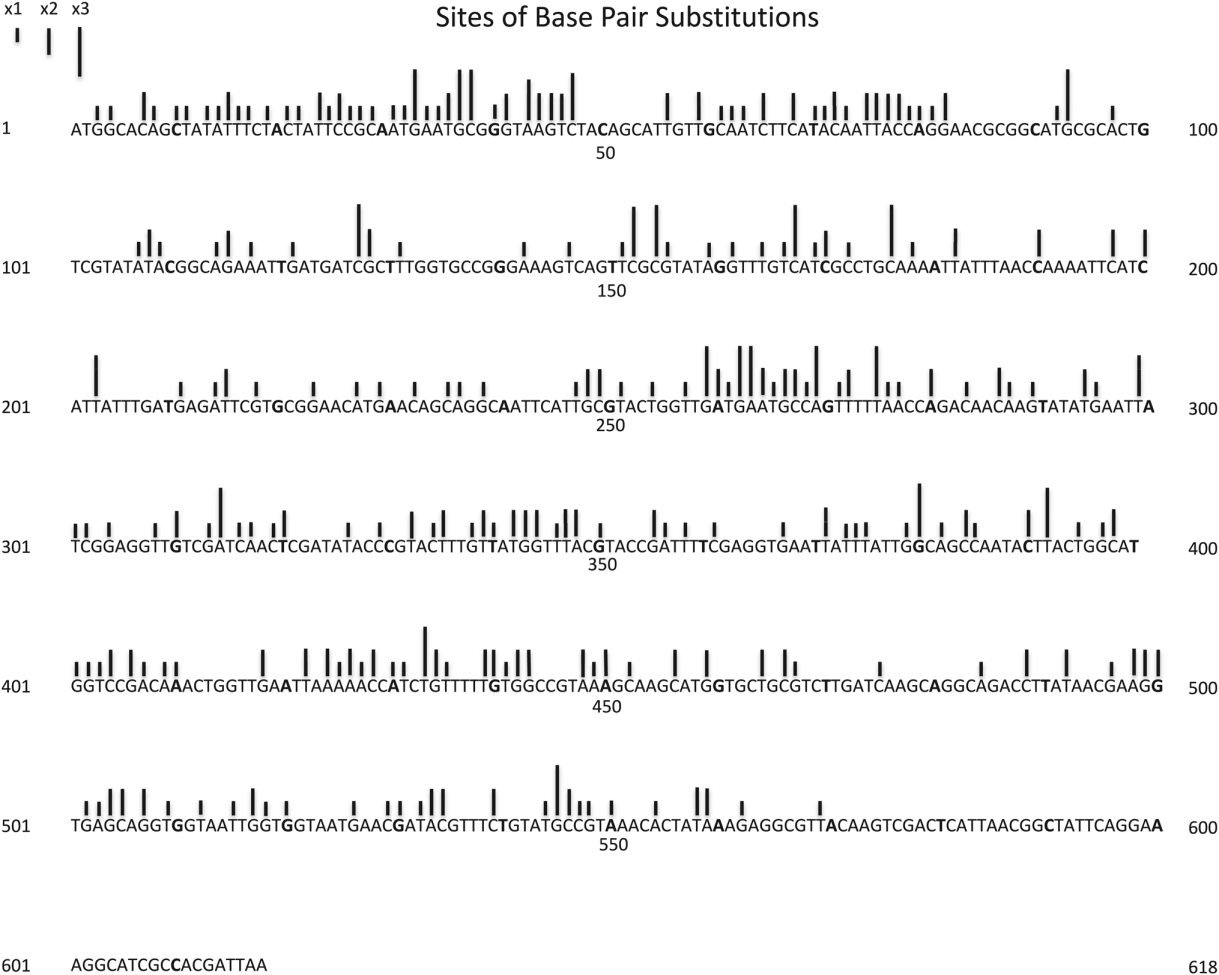
Density of the 245 sites found in the gene, independent of the number of occurrences found at each site. The height of each bar represents the number of different base pair substitutions found at that specific site (bp).

We treated strain BW25113 with five different mutagens; 2AP, CPT, 5AZ, EMS, and NQO, and plated on LB medium with AZT to find resistant mutants with defects in the *tdk* gene. We also plated *mutT*, *mutY*, *mutS*, and *mutD* derivatives of BW25113 on LB + AZT. AZT‐resistant colonies were purified and analyzed by PCR amplification and sequencing of the *tdk* region. Table 1 summarizes the mutation frequency of each condition. Figure 1 depicts the results for 1085 base substitution mutants. This includes 79 with 5AZ, 56 with 4NQO, 174 with EMS, 91 with 2AP, 170 with CPT, 178 with *mutT*, 48 with *mutS*, 171 with *mutY*, 24 with *mutD*, 82 with 2AP + 5AZ, and 12 spontaneous mutants. Here one can see a broad landscape of the results, with different mutagens having hotspots at different places. Taken together, these data allow us to construct a well saturated catalog of known mutational sites (Figure 2) and Tables 2, 3, 4, 5, 6, 7. To do this, we listed every mutation that we have detected in this study and in a previous study (Young et al. 2024), assorted according to the specific base substitution involved. We included each nonsense mutation that can be derived with a single base change, whether we have found the respective mutant in this particular collection or not. (Nonsense sites past the point near the very end of the gene where not even frameshifts have been detected are indicated with an asterisk but are not included in any counts or figures). We also included, in a few cases, base changes that generate the identical amino acid change that we have found to result in a mutant phenotype. For example, if we have detected one of the three base substitutions in the third position of the AUG codon, then we can list each of the other two changes at this position as available sites, since all three changes result in the Met to Ile change. The degree of saturation of possible sites can be approximated by asking how many of the possible nonsense sites we have found. For sites at G:C base pairs the degree of saturation is very high, as we have detected all 17 of the possible nonsense mutations arising from the G:C ‐>A:T transition, 28 of the possible 31 nonsense mutations arising from the G:C ‐>T:A transversion, and 8 of 9 nonsense mutations arising from the G:C ‐>C:G transversion. The degree of saturation of A:T appears less complete, as we have found 10 of 16 possible nonsense sites arising from the A:T ‐>C:G transversion, but only 8 of 30 arising from the A:T ‐>T:A transversion. The A:T ‐>G:C transition cannot be measured in this manner, as it cannot result in any of the three nonsense codons (Tables 3, 4, and 6).

**TABLE 2:** G:C ‐>A:T mutations.

Position	Triplet	AA change	Position	Triplet	AA change
128	CGC/GCG	Arg → His	127	TCG/CGA	Arg → Cys
38	GCG/CGC	Ala → Val	152	TCG/CGA	Ser → Leu
154	GCG/CGC	Arg → Cys	361	TCG/CGA	Arg → STOP
466	GCG/CGC	Arg → Cys	47	TCT/AGA	Ser → Phe
499	AGG/CCT	Gly → Ser	65	TCT/AGA	Ser → Phe
79	CCA/TGG	Gln → STOP	539	TCT/AGA	Ser → Phe
190	CCA/TGG	Gln → STOP	40	GGG/CCC	Gly → Ser
268	CCA/TGG	Gln → STOP	41	GGT/ACC	Gly → Asp
385	CCA/TGG	Gln → STOP	344	GGT/ACC	Gly → Asp
3	TGG/CCA	Met → Ile	402	GGT/ACC	Trp → STOP
343	TGG/CCA	Gly → Ser	500	GGT/ACC	Gly → Asp
401	TGG/CCA	Trp → STOP	7	ACA/TGT	Gln → STOP
459	TGG/CCA	Met → Ile	232	ACA/TGT	Gln → STOP
61	GCA/TGC	Gln → STOP	283	ACA/TGT	Gln → STOP
176	GCA/TGC	Ala → Val	286	ACA/TGT	Gln → STOP
235	GCA/TGC	Gln → STOP	571*	ACA/TGT	Gln → STOP
505	GCA/**TGC**	Gln → STOP	338	TGT/ACA	Cys → Tyr
37	TGC/GCA	Ala → Thr	434	TGT/ACA	Cys → Tyr
93	TGC/GCA	Met → Ile	440	TGT/ACA	Cys → Tyr
248	TGC/GCA	Cys → Tyr	115	AGA/TCT	Glu → Lys
266	TGC/GCA	Cys → Tyr	355	CGA/TCG	Asp → Asn
545	TGC/GCA	Cys → Tyr	155	CGT/ACG	Arg → His
33	TGA/TCA	Met → Ile	238	GGC/GCC	Ala → Thr
259	TGA/TCA	Asp → Asn	380	GGC/GCC	Gly → Asp
262	TGA/TCA	Glu → Lys	443	GGC/GCC	Gly → Asp
418	TGA/TCA	Glu → Lys	172	GCC/GGC	Pro → Ser
167	TCA/TGA	Ser → Leu	547	CCG/CGG	Arg → Cys
316	TCA/TGA	Gln → STOP	529	ACG/CGT	Arg → STOP
475	TCA/TGA	Gln → STOP	26	TCC/GGA	Ser → Phe
595*	TCA/TGA	Gln → STOP	404	TCC/GGA	Ser → Phe
334	ACT/AGT	Leu → Phe	**Total:**		59

* Not included in total mutation count.

**TABLE 3:** A:T ‐>G:C mutations.

Position	Triplet	AA change	Position	Triplet	AA change
265	ATG/CAT	Cys → Arg	559	TAA/TTA	Lys → Glu
544	ATG/CAT	Cys → Arg	25	TTC/GAA	Ser → Pro
254	CTG/CAG	Leu → Pro	247	TTG/CAA	Cys → Arg
433	CTG/CAG	Cys → Arg	46	GTC/GAC	Ser → Pro
269	CAG/CTG	Gln → Arg	166	GTC/GAC	Ser → Pro
280	CAG/CTG	Arg → Gly	403	GTC/GAC	Ser → Pro
169	ATC/GAT	Ser → Pro	263	GAA/TTC	Glu → Gly
301	ATC/GAT	Ser → Pro	320	CTC/GAG	Leu → Pro
260	GAT/ATC	Asp → Gly	43	TAA/TTA	Lys → Glu
314	GAT/ATC	Asp → Gly	35	AAT/ATT	Asn → Ser
335	CTT/AAG	Leu → Pro	272	TTT/AAA	Phe → Ser
203	TTA/TAA	Leu → Ser	503	GAG/CTC	Glu → Gly
299	TTA/TAA	Leu → Ser	77	TAC/GTA	Tyr → Cys
392	TTA/TAA	Leu → Ser	533	TAC/GTA	Tyr → Cys
Total:					28

**TABLE 4:** A:T ‐>C:G mutations.

Position	Triplet	AA change	Position	Triplet	AA change
8	CAG/CTG	Gln → Pro	346	TTT/AAA	Leu → Val
80	CAG/CTG	Gln → Pro	358	TTT/AAA	Phe → Val
236	CAG/CTG	Gln → Pro	374	TTT/AAA	Phe → Cys
269	CAG/CTG	Gln → Pro	97	CAC/GTG	Thr → Pro
382	CAG/CTG	Ser → Arg	554	CAC/GTG	His → Pro
395	CTG/CAG	Leu → Arg	320	CTC/GAG	Leu → Arg
433	CTG/CAG	Cys → Gly	34	GAA/TTC	Asn → His
464	CTG/CAG	Leu → Arg	263	GAA/TTC	Glu → Ala
76	TTA/TAA	Tyr → Asp	17	TTC/GAA	Phe → Cys
182	TTA/TAA	Leu → STOP	151	TTC/GAA	Ser → Ala
203	TTA/TAA	Leu → STOP	46	GTC/GAC	Ser → Ala
275	TTA/TAA	Leu → STOP	146	GTC/GAC	Val → Gly
299	TTA/TAA	Leu → STOP	257	GTT/AAC	Val → Gly
340	TTA/TAA	Tyr **→** Asp	271	GTT/AAC	Phe → Val
347	TTA/TAA	Leu → STOP	308	GTT/AAC	Val → Gly
371	TTA/TAA	Leu → STOP	339	GTT/AAC	Cys → Trp
375	TTA/TAA	Phe → Leu	14	TAT/ATA	Tyr → Ser
392	TTA/TAA	Leu → STOP	107	TAT/ATA	Tyr → Ser
422	TTA/TAA	Leu → STOP	13	ATA/TAT	Tyr → Asp
569	TTA/TAA	Leu → STOP	70	ATA/TAT	**Tyr →** Asp
584*	TTA/TAA	Leu → STOP	108	ATA/TAT	Tyr → STOP
43	TAA/TTA	Lys → Gln	326	ATA/TAT	Ile → Arg
276	TAA/TTA	Leu → Phe	492	ATA/TAT	Tyr → STOP
448	TAA/TTA	Lys → Gln	532	ATA/TAT	Tyr → Asp
227	CAT/ATG	His → Pro	558	ATA/TAT	Tyr → STOP
32	ATG/CAT	Met → Arg	214	GAT/ATC	Ile → Leu
36	ATG/CAT	Asn → Lys	260	GAT/ATC	Asp → Ala
261	ATG/CAT	Asp → Glu	314	GAT/ATC	Asp → Ala
294	ATG/CAT	Tyr → STOP	356	GAT/ATC	Asp → Ala
342	ATG/CAT	Tyr → STOP	56	TTG/CAA	Leu → Trp
31	AAT/ATT	Met → Leu	59	TTG/CAA	Leu → Trp
63	AAT/ATT	Gln → His	439	TTG/CAA	Cys → Gly
264	AAT/ATT	Glu → Asp	62	CAA/TTG	Gln → Pro
15	ATT/AAT	Tyr → STOP	287	CAA/TTG	Gln → Pro
24	ATT/AAT	Tyr → STOP	317	CAA/TTG	Gln → Pro
75	ATT/AAT	Asn → Lys	71	TAC/GTA	Tyr → Ser
215	ATT/AAT	Ile → Ser	77	TAC/GTA	Tyr → Ser
335	CTT/AAG	Leu → Arg	109	TAC/GTA	Thr → Pro
44	AAG/CTT	Lys → Thr	348	TAC/GTA	Leu → Phe
450	AAG/CTT	Lys → Asn	533	TAC/GTA	Tyr → Ser
498	AAG/CTT	Glu → Asp	332	GTA/TAC	Val → Gly
117	AAA/TTT	Glu → Asp	277	AAC/GTT	Thr → Pro
410	AAA/TTT	Lys → Thr	427	AAC/GTT	Thr → Pro
424	AAA/TTT	Lys → Gln	11	CTA/TAG	Leu → Arg
425	AAA/TTT	Lys → Thr	19	CTA/TAG	Tyr → Asp
426	AAA/TTT	Lys → Asn	22	CTA/TAG	Tyr → Asp
449	AAA/TTT	Lys → Thr	431	ATC/GAT	Ile → Ser
16	TTT/AAA	Phe → Val	509	GTG/CAC	Val → Gly
131	TTT/AAA	Phe → Cys	407	GAC/GTC	Asp → Ala
272	TTT/AAA	Phe → Cys	Total:		98

* Not included in total mutation count.

**TABLE 5:** G:C ‐>T:A mutations.

Position	Triplet	AA change	Position	Triplet	AA change
499	AGG/CCT	Gly → Cys	310	TGT/ACA	Val → Phe
507	AGG/CCT	Gln → His	434	TGT/ACA	Cys → Phe
488	CCT/AGG	Pro → His	440	TGT/ACA	Cys → Phe
33	TGA/TCA	Met → Ile	286	ACA/TGT	Thr → Lys
211	TGA/TCA	Glu → STOP	41	GGT/ACC	Gly → Val
229	TGA/TCA	Glu → STOP	161	GGT/ACC	Gly → Val
259	TGA/TCA	Asp → Tyr	344	GGT/ACC	Gly → Val
262	TGA/TCA	Glu → STOP	518	GGT/ACC	Gly → Val
295	TGA/TCA	Glu → STOP	78	ACC/GGT	Tyr → STOP
367	TGA/TCA	Glu → STOP	428	ACC/GGT	Thr → Asn
418	TGA/TCA	Glu → STOP	82	GGA/TCC	Glu → STOP
502	TGA/TCA	Glu → STOP	223	GGA/TCC	Glu → STOP
526	TGA/TCA	Glu → STOP	304	GGA/TCC	Glu → STOP
68	TCA/TGA	Ser → STOP	598*	GGA/TCC	Glu → STOP
167	TCA/TGA	Ser → STOP	404	TCC/GGA	Ser → Tyr
197	TCA/TGA	Ser → STOP	72	ACA/TGT	Glu → STOP
200	TCA/TGA	Ser → STOP	115	AGA/TCT	Glu → STOP
343	TGG/CCA	Gly → Cys	562	AGA/TCT	Glu → STOP
442	TGG/CCA	Gly → Cys	47	TCT/AGA	Ser → Tyr
459	TGG/CCA	Met → Ile	65	TCT/AGA	Ser → Tyr
517	TGG/CCA	Gly → Cys	539	TCT/AGA	Ser → Tyr
520	TGG/CCA	Gly → Cys	504	AGC/GCT	Glu → Asp
190	CCA/TGG	Gln → Lys	267	GCC/GGC	Cys → STOP
127	TCG/CGA	Arg → Ser	384	GCC/GGC	Ser → Arg
152	TCG/CGA	Ser → STOP	546	GCC/GGC	Cys → STOP
170	TCG/CGA	Ser → STOP	380	GGC/GCC	Gly → Val
302	TCG/CGA	Ser → STOP	443	GGC/GCC	Gly → Val
113	GCA/TGC	Ala → Glu	45	AGT/ACT	Lys → Asn
176	GCA/TGC	Ala → Glu	289	AGT/ACT	Val → Leu
398	GCA/TGC	Ala → Glu	21	ACT/AGT	Tyr → STOP
452	GCA/TGC	Ala → Glu	319	ACT/AGT	Leu → Ile
456	GCA/TGC	Ser → Arg	390	ACT/AGT	Tyr → STOP
313	CGA/TCG	Asp → Tyr	534	ACG/CGT	Tyr → STOP
355	CGA/TCG	Asp → Tyr	329	CCC/GGG	Pro → His
406	CGA/TCG	Asp → Tyr	38	GCG/CGC	Ala → Glu
496	CGA/TCG	Glu → STOP	154	GCG/CGC	Arg → Ser
37	TGC/GCA	Ala → Ser	249	GCG/CGC	Cys → STOP
248	TGC/GCA	Cys → Phe	466	GCG/CGC	Arg → Ser
266	TGC/GCA	Cys → Phe	28	CGC/GCG	Ala → Ser
545	TGC/GCA	Cys → Phe	128	CGC/GCG	Arg → Leu
93	TGC/GCA	Met → Ile	**Total:**		81

* Not included in total mutation count.

**TABLE 6:** A:T ‐>T:A mutations.

Position	Triplet	AA change	Position	Triplet	AA change
269	CAG/CTG	Gln → Leu	450	AAG/CTT	Lys → **Asn**
280	CAG/CTG	Arg → STOP	498	AAG/CTT	Glu → Asp
484	CAG/CTG	Arg → STOP	348	TAC/GTA	Leu → Phe
433	CTG/CAG	Cys → Ser	56	TTG/CAA	Leu → STOP
464	CTG/CAG	Leu → Gln	59	TTG/CAA	Leu → STOP
15	ATT/AAT	Tyr → STOP	164	TTG/CAA	Leu → STOP
24	ATT/AAT	Tyr → STOP	439	TTG/CAA	Cys → Ser
75	ATT/AAT	Asn → Lys	73	CAA/TTG	Asn → Tyr
215	ATT/AAT	Ile → Asn	409	CAA/TTG	Lys → STOP
373	ATT/AAT	Phe → Ile	36	ATG/CAT	Asn → Lys
515	ATT/AAT	Ile → Asn	92	ATG/CAT	Met → Lys
264	AAT/ATT	Glu → Asp	294	ATG/CAT	Tyr → STOP
76	TTA/TAA	Tyr → Asn	342	ATG/CAT	Tyr → STOP
182	TTA/TAA	Leu → STOP	430	CAT/ATG	Ile → Phe
203	TTA/TAA	Leu → STOP	251	GTA/TAC	Val → Glu
275	TTA/TAA	Leu → STOP	332	GTA/TAC	Val → Glu
299	TTA/TAA	Leu → STOP	512	GTA/TAC	Val → Glu
347	TTA/TAA	Leu → STOP	441	GTG/CAC	Cys → STOP
371	TTA/TAA	Leu → STOP	142	GAA/TTC	Lys → STOP
376	TTA/TAA	Phe → Leu	263	GAA/TTC	Glu → Val
392	TTA/TAA	Leu → STOP	108	ATA/TAT	Tyr → STOP
422	TTA/TAA	Leu → STOP	492	ATA/TAT	Tyr → STOP
569*	TTA/TAA	Leu → STOP	558	ATA/TAT	Tyr → STOP
584*	TTA/TAA	Leu → STOP	314	GAT/ATC	Asp → Val
43	TAA/TTA	Lys → STOP	339	GTT/AAC	Cys → STOP
276	TAA/TTA	Leu → Phe	435	GTT/AAC	Cys → STOP
448	TAA/TTA	Lys → STOP	178	AAA/TTT	Lys → STOP
550	TAA/TTA	Lys → STOP	424	AAA/TTT	Lys → STOP
559	TAA/TTA	Lys → STOP	426	AAA/TTT	Lys → Asn
44	AAG/CTT	Lys → Met	**Total**		58

* Not included in total mutation count.

**TABLE 7:** G:C ‐>C:G mutations.

Position	Triplet	AA change	Position	Triplet	AA change
218	CGT/ACG	Arg → Pro	390	ACT/AGT	Tyr → STOP
350	CGT/ACG	Arg → Pro	7	ACA/TGT	Gln → Glu
467	CGT/ACG	Arg → Pro	72	ACA/TGT	Tyr → STOP
548	CGT/ACG	Arg → Pro	78	ACC/GGT	Tyr → STOP
534	ACG/CGT	Tyr → STOP	428	ACC/GGT	Thr → Ser
267	GCC/GGC	Cys → Trp	37	TGC/GCA	Ala → Pro
384	GCC/GGC	Cys → Trp	93	TGC/GCA	Met → Ile
546	GCC/GGC	Cys → Trp	545	TGC/GCA	Cys → Ser
4	GGC/GCC	Ala → Pro	406	CGA/TCG	Asp → His
380	GGC/GCC	Gly → Ala	530	CGA/TCG	Arg → Pro
397	GGC/GCC	Ala → Pro	379	TGG/CCA	Gly → Arg
68	TCA/TGA	Ser → STOP	442	TGG/CCA	Gly → Arg
167	TCA/TGA	Ser → STOP	459	TGG/CCA	Met → Ile
197	TCA/TGA	Ser → STOP	517	TGG/CCA	Gly → Arg
200	TCA/TGA	Ser → STOP	310	TGT/ACA	Val → Leu
581*	TCA/TGA	Ser → STOP	38	GCG/CGC	Ala → Gly
33	TGA/TCA	Met → Ile	154	GCG/CGC	Arg → Gly
121	TGA/TCA	Asp → His	249	GCG/CGC	Cys → Trp
259	TGA/TCA	Asp → His	45	AGT/ACT	Lys → Asn
262	TGA/TCA	Glu → Gln	47	TCT/ACA	Ser → Cys
127	TCG/CGA	Arg → Gly	488	CCT/AGG	Pro → Arg
152	TCG/CGA	Ser → Trp	500	GGT/ACC	Gly → Ala
170	TCG/CGA	Ser → Trp	507	AGG/CCT	Gln → His
29	GCA/TGC	Ala → Gly	10	GCT/AGC	Leu → Val
398	GCA/TGC	Ala → Gly	504	AGC/GCT	Glu → Asp
456	GCA/TGC	Ser → Arg	82	GGA/TCC	Glu → Gln
505	GCA/TGC	Gln → Glu	26	TCC/GGA	Ser → Cys
21	ACT/AGT	Tyr → STOP	Total		54

* Not included in total mutation count.

The current catalog consists of 378 different mutations at 245 different sites (bp). Figure 2 focuses on the sites without regard to the number of occurrences found in the sample, to allow a better visualization of the density of available sites throughout the gene. (Figure 2 and subsequent figures do not include several mutations found as part of double mutations, but that would result in silent changes themselves). Again, all the sites in Figure 2 are base pairs at which we have demonstrated that base substitutions cause a defective TDK protein that results in AZT resistance. The tables list each site arranged by the resulting type of base substitution and the nearest neighbors.

### 3.2 Finding Hot Regions Within the Gene

**FIGURE 3:**
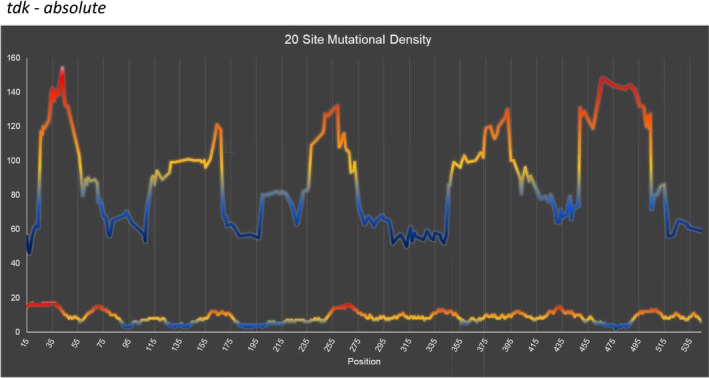
Mutation density, Each point on the top portion of the graph llustrates total mutations occurring in a 20 site interval with the midpoint at the point shown. Sites are defined as a base pair at which mutation(s) have been detected. The red‐blue color scheme represents hotter (red) vs. colder (blue) regions of the gene. On the bottom portion of the graph is the density of available sites.

**FIGURE 4:**
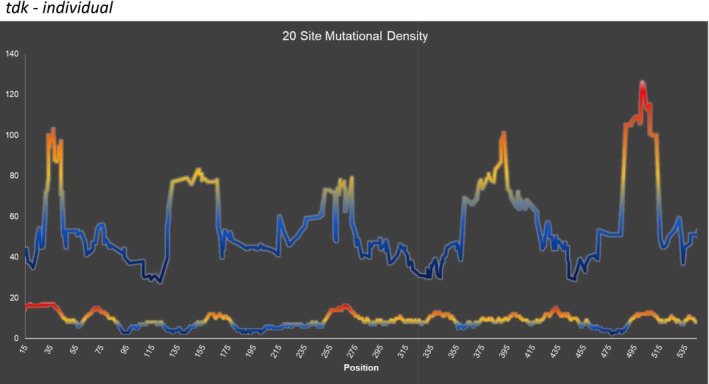
Mutation density, Each point on the top portion of the graph illustrates total mutations occurring in a 20 site interval with the midpoint at the point shown. Here, each type of mutation counts as an additional site; so if all 3 types of AT or GC mutations occur at a specific site, this would be considered as 3 distinct sites. The red‐blue color scheme represents hotter (red) versus colder (blue) regions of the gene. On the bottom portion of the graph is the density of available sites.

As we have done for the *thyA* gene (Mashiach et al. 2021), we can superimpose the mutations found (Figure 1), with the density of known mutational sites (Figure 2) to reveal regions of high or low mutability. Thus we chart the total number of mutations found in a rolling interval, the intervals being defined not by distance but by a set number of available sites. This is somewhat analogous to looking at the G:C to A:T ratio at a rolling interval of 20 or 30 base pairs, except that the intervals are defined differently. Figures 3 and 4 show the results for intervals of 20 sites. We can define a site as a base pair at which mutations have been found that result in an AZT‐resistant mutant, independent of whether we have found one, two, or three different base changes at that site (“Absolute,” Figure 3). Or, we can define a “site” for each mutation, so if a base pair has been found to have three different changes detected we would designate that as three sites (“Individual,” Figure 4). Clearly, both methods of determining available “sites” give very similar curves. These show that there are mutationally prone regions (MPRs) in five places that are separated by regions of lower mutability. In Figures 3 and 4 we have also shown the density of sites at the bottom of each set of curves. This allows us to ask whether the peaks in the upper part of the figure always correspond to the regions of high site density, so the apparent MPRs are only a function of high site density and not necessarily a reflection of higher mutability of that region. One can see that this is not the case, as some peaks of density are not aligned with peaks of high mutability, and some peaks of high mutability are not aligned with peaks of high density. As described above, the degree of saturation of possible sites at GC base pairs is higher than for AT base pairs. Redoing Figures 3 and 4 using only GC sites gives the same picture as shown in Figures 3 and 4 here (data not shown). Moreover, doing the same figures with only the AT sites still gives three prominent peaks.

**FIGURE 5:**
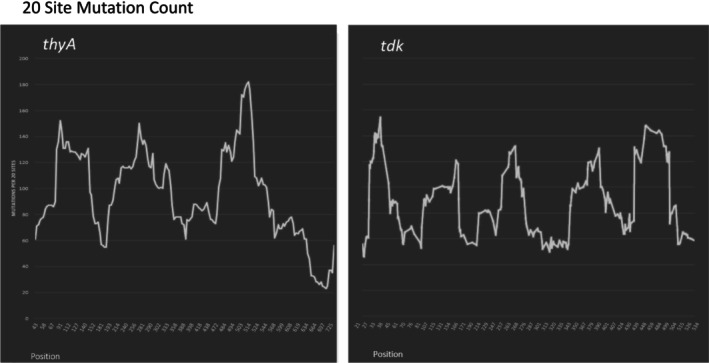
A compilation of the results shown here in Figure (right panel) with those in the *thyA* gene we obtained previously (Mashiach et al. 2021; left panel).

We can align the results from this study together with those of our similar analysis in the *thyA* gene (Mashiach et al. 2021). Placed side by side, the two studies, each involving a similar number of mutations, reveal 8 MPR regions of somewhat similar heights and sizes (Figure 5).

## 4 Discussion

Deciphering all the rules governing hotspots has proved elusive. Although “indels” (short sequence additions or deletions often leading to frameshifts) clearly occur preferentially at repeated sequence elements (e.g., Streisinger et al. 1966; Farabaugh et al. 1978; Pribnow et al. 1981), base substitution hotspots seem to follow a more complicated set of preferences. The nearest neighbor sequences, meaning the base directly 5' and the base directly 3' to the base undergoing change, often exert an important influence on mutation rates (Coulondre and Miller 1977; Miller 1985; Pienkowska et al. 1993; Foster et al. 2018; Fernandez et al. 2020). In fact, this has been instrumental in assigning mutational “signatures” to different classes of mutagens in higher cell cultures after genomic sequencing (e.g., Phillips 2018). However, there are multiple instances of bases with the same nearest neighbors having dramatically different mutation rates (e.g., Miller 1985; Garibyan et al. 2003; see Discussion in Fernandez et al. 2020). We have built two systems to examine whether certain regions of the gene are more mutation prone than others, designating these regions as MPRs (mutation prone regions). In order to do this, we construct a catalog of all the mutations we can define that result in a mutant phenotype that we can select for, as detailed in Results. The value of such a catalog is that mutagenic specificities and preferences can be assayed against an array of known sites. For instance, in *tdk*, we have defined 81 specific sites at which a G:C –> T:A mutation results in an AZT resistant mutant. These are listed together with the nearest neighbors (e.g., Table 5). Most important, one can record all the sites at which there are zero occurrences in any particular mutant collection. This cannot be done without a catalog of known sites. We use approximately 1100 mutations from multiple experiments with different mutagens or mutator strains. We then chart all the known sites and look at the number of mutations in this large collection in each interval of 20 or 25 known mutational sites in a continuous plot moving throughout the gene. This is analogous to plots of AT richness in intervals of 20 or 25 bp moving along the gene length. We first used this approach with the *thyA* gene of *E. coli* and defined 3 distinct MPRs (Mashiach et al. 2021). Mutations occurred in these regions 3–8 fold more frequently on a per equal number of sites basis than mutations in other regions. Because not every mutator or mutagen may not follow the same rules, this 3–8 fold effect might be somewhat muted. Therefore, we sought to examine the distribution of mutations from a single mutagen and also sought a second system to extend the analysis to a second gene to possibly increase the number of mutationally prone regions detected.

We based our second system on the *tdk* gene in *E. coli* , that encodes thymidylate deoxykinase. Mutants resistant to AZT have mutations in ***tdk*** that result in an inactive enzyme. The system has advantages over the *thyA* system for detecting base substitution mutations in that mutants appear on LB plates with AZT after 18–24 h, and the spontaneous background is devoid of distinctive hotspots, unlike the *thyA* gene (see Viswanathan et al. 2000). The gene length being only 618 base pairs affords easy PCR and sequencing with a single primer pair. We have reported the use of the *tdk* system to examine the distribution of mutations induced by cisplatin (CPT), and shown that a specific hotspot occurs only in a certain region of the gene and not in at least three other regions of the gene despite the availability of known mutational sites (Young et al. 2024). Here we show 5 mutation prone regions in *tdk* (Figures 3 and 4) and align these together with the three peaks in *thyA* (Figure 5). The array of 8 peaks seen in Figure 5 makes a convincing case for the concept that some regions of genes are more mutationally prone (MPRs) than other regions. This is not due to regions encoding critical residues for enzymatic activity, but rather due to these regions being more susceptible to mutational change. Why has this not been reported previously? First, the way we have chosen to show this depends on first making a catalog of all the observable mutations emanating from a series of mutagens and mutators. Then, one can look at the number of mutations found in a large sample that is normalized to the number of available mutational sites. This type of analysis had not been extensively carried out, to our knowledge. Second, for the total mutations from a pool of close to 1100 mutations arising from 9 different treatments or mutator strains, the effects are not all or nothing. These effects range from 2 to 3 fold to 8 fold. However, when certain specific mutagens are studied, such as cisplatin, the effects can be dramatic. Thus, all 21 of the cisplatin‐induced G:C ‐>T:A mutations in *tdk* that occur at 5'‐CGA‐3' sequences are at one site, at bp 496, and none are at any of the three other sites (bp 313, 355, 406) that are available in the gene and at which we have detected G:C ‐>T:A mutations with other mutagens (Young et al. 2024). Only the 496 site is in a MPR, as the other three sites are not.

Why are some regions of the gene more mutation prone? Could this emanate from the “quasi‐nucleoid” structure of the chromosome? Verna, Qian, and Adhya (Verma et al. 2019) have extensively reviewed studies of the organization of the *E. coli* chromosome (Kahramanoglou et al. 2011; Prieto et al. 2012; Johnson et al. 2021; Lioy et al. 2018), which is highly condensed and folded into loops. The resulting “topological domains” can be altered and can affect gene expression. Nucleoid‐associated proteins, such as HU, FIS, and IHF, cover parts of the chromosome and condense the DNA. These three proteins together cover 18% of the chromosome (Johnson et al. 2021). Perhaps regions of low mutability are more covered than regions of high mutability. Although carrying out studies such as this and that of Mashiach et al. (2021) is laborious to repeat in a set of mutants deficient in different nucleoid proteins, it is feasible to examine the effects of such mutants on, for instance, the frequency and distribution of cisplatin‐induced mutants in *tdk*, as reported in Young et al. (2024). Such work is in progress. Could special sequences play a role in determining hotspot regions? There are two GATC sequences in *tdk* that signal Dam methylation at one adenine on each strand as a guide to mismatch repair (e.g., Barras and Marinus 1989; Lobner‐Olesen et al. 2005); one at bp 313–316 and the other at bp 472–475. However, whereas the latter is in a region of high mutational density, the former is in a region of very low mutational density. There are no perfect 8 bp Chi‐sites in *tdk* that signal recombination via the RecBCD pathway (Myers and Stahl 1994), although there is a sequence from bp 504–511 that is 1 bp away from the true site. This is in a region of high mutational density, and some imperfect Chi‐sites retain some activity of a full Chi‐site (Cheng and Smith 1984). There are no other near Chi sites for the other 4 mutationally prone regions. Also, examination of the GC or AT richness of subregions of the gene fails to show a correlation with mutationally prone regions.

## 5 Conclusion

Therefore, we can attribute the differences in base substitution mutation rates at sites with the identical nearest neighbors to the crucial element in determining which sites are true “hotspots,” namely being in a particular region of the gene. The larger implications are that mutation frequency is significantly influenced by the structure of regions of DNA, and this points to the value of future studies of what these structures are and how changes in these structures affect mutability.

## Author Contributions

Jeffrey H. Miller designed the study. Katherine Douglas, Dana Sorensen, Arnav Saud, Ananya Sridharan, Mallika Mathew, Jyotsna Hiranandani, Ava Gonick, Courtney Young, Kelly Nguyen, and Nhu Phi performed the experiments and generated the figures. Jeffrey H. Miller wrote the manuscript. All authors reviewed the submitted version of the manuscript.

## Conflicts of Interest

The authors declare no conflicts of interest.

## Data Availability

Research data are not shared.

## References

[em70021-bib-0001] Ang, J. , L. Y. Song , S. D'Souza , et al. 2016. “Mutagen Synergy: Hypermutability Generated by Specific Pairs of Base Analogs.” Journal of Bacteriology 198: 2776–2783. 27457718 10.1128/JB.00391-16 PMC5038009

[em70021-bib-0002] Baba, T. , T. Ara , M. Hasegawa , et al. 2006. “Construction of *Escherichia Coli* K12 In‐Frame Single‐Gene Knockout Mutants: The KEIO Collection.” Molecular Systems Biology 2: 2006.0008. 10.1038/msb4100050 PMC1681482 16738554

[em70021-bib-0003] Barras, F. , and M. G. Marinus . 1989. “The Great GATC: DNA Methylation in *E. coli* .” Trends in Genetics 5: 139–143. 2667217 10.1016/0168-9525(89)90054-1

[em70021-bib-0004] Benzer, S. 1961. “On the Topography of the Genetic Fine Structure.” Proceedings of the National Academy of Sciences of the United States of America 47: 403–415. 16590840 10.1073/pnas.47.3.403 PMC221592

[em70021-bib-0005] Bertenyi, K. K. A. , and I. B. Lambert . 1996. “The Mutational Specificity of Furazolidone in the *lacI* Gene of *Escherichia coli* .” Mutation Research, Fundamental and Molecular Mechanisms of Mutagenesis 357: 199–208. 8876695 10.1016/0027-5107(96)00102-9

[em70021-bib-0006] Cheng, K. C. , and G. R. Smith . 1984. “Recombinational Hotspot Activity of chi‐Like Sequences.” Journal of Molecular Biology 180: 373–377. 10.1016/s0022-2836(84)80009-1 6239928

[em70021-bib-0007] Coulondre, C. , and J. H. Miller . 1977. “Genetic Studies of the Lac Repressor. IV. Mutagenic Specificity in the lacI Gene of *Escherichia coli* .” Journal of Molecular Biology 117: 577–606. 416218 10.1016/0022-2836(77)90059-6

[em70021-bib-0008] Datsenko, K. A. , and B. L. Wanner . 2000. “One‐Step Inactivation of Chromosomal Genes in *Escherichia coli* K‐12 Using PCR Products.” Proceedings of the National Academy of Sciences of the United States of America 97: 6640–6645. 10829079 10.1073/pnas.120163297 PMC18686

[em70021-bib-0009] Dixon, W. J. , and F. J. Massey Jr. . 1969. Introduction to Statistical Analysis. McGraw‐Hill.

[em70021-bib-0010] Farabaugh, P. J. , U. Schmeissner , M. Hofer , and J. H. Miller . 1978. “Genetic Studies of the *Lac* Repressor. VII. On the Molecular Nature of Spontaneous Hotspots in the *lacI* Gene of *Escherichia coli* .” Journal of Molecular Biology 126: 847–863. 370408 10.1016/0022-2836(78)90023-2

[em70021-bib-0011] Fernandez, K. , S. D'Souza , J. J. Ahn , et al. 2020. “Mutations Induced by Bleomycin, 4‐Nitroquinoline‐1‐Oxide, and Hydrogen Peroxide in the *rpoB* Gene of *Escherichia coli*: Perspective on Mutational Hotspots.” Mutation Research, Fundamental and Molecular Mechanisms of Mutagenesis 821: 111702. 32422468 10.1016/j.mrfmmm.2020.111702

[em70021-bib-0012] Foster, P. L. , B. A. Niccum , E. M. Popodi , et al. 2018. “Determinants of Base Substitution Patterns Revealed by Whole Genome Sequencing of DNA Mismatch Repair Defective *Escherichia coli* .” Genetics 209: 1029–1042. 29907647 10.1534/genetics.118.301237 PMC6063221

[em70021-bib-0013] Garibyan, L. , T. Huang , T. M. Kim , et al. 2003. “Use of the *rpoB* Gene to Determine the Specificity of Base Substitution Mutations on the *Escherichia coli* Chromosome.” DNA Repair 2: 593–608. 12713816 10.1016/s1568-7864(03)00024-7

[em70021-bib-0014] Hatahet, Z. , M. Zhou , L. J. Reha‐Krantz , S. W. Morrical , and S. S. Wallace . 1998. “In Search of a Mutational Hotspot.” Proceedings of the National Academy of Sciences of the United States of America 95: 8561–8886. 10.1073/pnas.95.15.8556 PMC21114 9671716

[em70021-bib-0015] Horsfall, M. J. , A. J. E. Gorden , P. A. Burns , M. Zielenska , G. M. E. van der Vliet , and B. W. Glickman . 1990. “Mutational Specificity of Alkylating Agents and the Influence of DNA Repair.” Environmental and Molecular Mutagenesis 15: 107–122. 2407530 10.1002/em.2850150208

[em70021-bib-0016] Johnson, R. C. , L. M. Johnson , J. W. Schmidt , and J. F. Gardner . 2021. “Major Nucleoid Proteins in the Structure and Function of the *Escherichia coli* Chromosome.” In The Bacterial Chromosome, edited by N. P. Higgins , 65–132. ASM Press.

[em70021-bib-0017] Kahramanoglou, C. , A. S. N. Seshasayee , A. I. Rieto , et al. 2011. “Direct and Indirect Effects of H‐NS and Fis on Global Gene Expression Control in *Escherichia Coli* .” Nucleic Acids Research 39: 2073–2091. 21097887 10.1093/nar/gkq934 PMC3064808

[em70021-bib-0018] Lawrence, M. S. , P. Stojanov , P. Polak , et al. 2013. “Mutational Heterogeneity in Cancer and the Search for New Cancer‐Associated Genes.” Nature 499, no. 7457: 214–218. 23770567 10.1038/nature12213 PMC3919509

[em70021-bib-0019] Lioy, V. S. , A. Cournac , M. Marbouty , et al. 2018. “Multiscale Structuring of the *E. coli* Chromosome Nucleoid‐Associated and Condensing Proteins.” Cell 172: 771–783. 29358050 10.1016/j.cell.2017.12.027

[em70021-bib-0020] Lobner‐Olesen, A. , O. Skovgaard , and M. G. Marinus . 2005. “Dam Methylation: Coordinating Cellular Processes.” Current Opinion in Microbiology 8: 154–160. 15802246 10.1016/j.mib.2005.02.009

[em70021-bib-0021] Mashiach, D. , E. M. Bacasen , S. Singh , et al. 2021. “Enhanced Characterization of the thyA System for Mutational Analysis in Escherichia Coli: Defining Mutationally “Hot” Regions of the Gene.” Mutation Research, Fundamental and Molecular Mechanisms of Mutagenesis 823: 111754. 34091127 10.1016/j.mrfmmm.2021.111754

[em70021-bib-0022] Miller, J. H. 1972. Experiments in Molecular Genetics. Cold Spring Harbor Laboratory Press.

[em70021-bib-0023] Miller, J. H. 1985. “Mutagenic Specificity of Ultraviolet Light.” Journal of Molecular Biology 182: 45–68. 3923204 10.1016/0022-2836(85)90026-9

[em70021-bib-0024] Miller, J. H. 1992. A Short Course in Bacterial Genetics: A Laboratory Manual and Handbook for Escherichia Coli and Related Bacteria. Cold Spring Harbor Laboratory Press.

[em70021-bib-0025] Myers, R. S. , and F. W. Stahl . 1994. “Chi and the RecBCD Enzyme of *Escherichia coli* .” Annual Revue of Genetics 28: 49–70. 10.1146/annurev.ge.28.120194.000405 7893137

[em70021-bib-0026] Nilsen, H. , P. Yazdankhah , I. Eftedal , and H. E. Krokan . 1995. “Sequence Specificity for Removal of Uracil From U‐A Pairs and U‐G Mismatches by Uracil‐DNA Glycosylase From *Eschericia coli*, and Correlation With Mutational Hotspots.” FEBS Letters 362: 205–209. 7720873 10.1016/0014-5793(95)00244-4

[em70021-bib-0027] Phillips, D. H. 2018. “Mutational Spectra and Mutational Signatures: Insights Into Cancer Aetiology and Mechanisms of DNA Damage and Repair.” DNA Repair 71: 6–11. 30236628 10.1016/j.dnarep.2018.08.003 PMC6219445

[em70021-bib-0028] Pienkowska, M. , B. W. Glickman , A. Ferreira , M. Anderson , and M. Zielenska . 1993. “Large‐Scale Mutational Analysis of EMS‐Mutation in the *lacI* Gene of *Escherichia coli* .” Mutation Research 288: 123–131. 7686256 10.1016/0027-5107(93)90214-z

[em70021-bib-0029] Piraino, S. W. , and S. J. Furney . 2017. “Identification of Coding and Non‐Coding Mutational Hotspots in Cancer Genomes.” BMC Genomics 18: 17. 28056774 10.1186/s12864-016-3420-9 PMC5217664

[em70021-bib-0030] Pribnow, D. , D. C. Sigurdson , L. Gold , et al. 1981. “rII Cistrons of Bacteriophage T4: DNAsequences Around the Inter Cistronic Divide and Positions of Genetic Landmarks.” Journal of Molecular Biology 149: 337–376. 6273585 10.1016/0022-2836(81)90477-0

[em70021-bib-0031] Prieto, A. I. , C. Kahramanoglou , R. M. Ali , G. M. Fraser , A. S. N. Seshasayee , and N. M. Luscombe . 2012. “Genomic Analysis of DNA Binding and Gene Regulation by Homologous Nucleoid‐Associated Protein IHF and HU in *Escherichia coli* .” Nucleic Acids Research 40: 3524–3537. 22180530 10.1093/nar/gkr1236 PMC3333857

[em70021-bib-0032] Rodriguez, H. , and E. L. Loechler . 1991. “Mutational Specificityof the (+)‐anti‐Diol Epoxide of Benzo[a]Pyrene in a *supF* Gene of an *Escherichia coli* Plasmid: DNA Sequence Context Influences Hotspots, Mutagenic Specificity and the Extent of SOS Enhancement of Mutagenesis.” Carcinogenesis 14: 373–383. 10.1093/carcin/14.3.373 8453713

[em70021-bib-0033] Shee, C. , J. L. Gibson , and S. M. Rosenberg . 2012. “Two Mechanisms Produce Mutational Hotspots at DNA Breaks in *Escherichia coli* .” Cell Reports 2: 714–721. 23041320 10.1016/j.celrep.2012.08.033 PMC3607216

[em70021-bib-0034] Streisinger, G. , Y. Okada , J. Emrich , et al. 1966. “Frameshift Mutations and the Genetic Code.” Cold Spring Harbor Symposia on Quantitative Biology 31: 77–86. 5237214 10.1101/sqb.1966.031.01.014

[em70021-bib-0035] Verma, S. C. , Z. Qian , and S. I. Adhya . 2019. “Architecture of the *Escherichia coli* Nucleoid.” PLoS Genetics 15, no. 21: e1008456. 31830036 10.1371/journal.pgen.1008456 PMC6907758

[em70021-bib-0036] Viswanathan, M. , J. J. Lacirignola , R. L. Hurley , and S. T. Lovett . 2000. “A Novel Mutational Hotspot in a Natural Quasipalindrom in *Escherichia coli* .” Journal of Molecular Biology 302: 553–564. 10986118 10.1006/jmbi.2000.4088

[em70021-bib-0037] Young, C. , M. Lee , Z. Ge , et al. 2024. “Anatomy of a Hotspot: Cisplatin Hotspots in the *Tdk* Gene of *Escherichia coli* .” Environmental and Molecular Mutagenesis 65, no. 9: 338–350. 39387394 10.1002/em.22635 PMC11603525

